# Functional Sr_0.5_Ba_0.5_Sm_0.02_Fe_11.98_O_4_/*x*(Ni_0.8_Zn_0.2_Fe_2_O_4_) Hard–Soft Ferrite Nanocomposites: Structure, Magnetic and Microwave Properties

**DOI:** 10.3390/nano10112134

**Published:** 2020-10-27

**Authors:** Norah A. Algarou, Yassine Slimani, Munirah A. Almessiere, Ali Sadaqat, Alex V. Trukhanov, Mohammad A. Gondal, Abbas S. Hakeem, Sergei V. Trukhanov, Maksim G. Vakhitov, Denis S. Klygach, Ayyar Manikandan, Abdulhadi Baykal

**Affiliations:** 1 Department of Biophysics, Institute for Research and Medical Consultations (IRMC), Imam Abdulrahman Bin Faisal University, P.O. Box 1982, Dammam 31441, Saudi Arabia; nalgarou@iau.edu.sa; 2 Department of Physics, College of Science, Imam Abdulrahman Bin Faisal University, P.O. Box 1982, Dammam 31441, Saudi Arabia; 3 Department of Mechanical and Energy Engineering, College of Engineering, Imam Abdulrahman Bin Faisal University, P.O. Box 1982, Dammam 31441, Saudi Arabia; sadali@iau.edu.sa; 4 Department of Electronic Materials Technology, Institute of New Materials and Nanotechnology, National University of Science and Technology MISiS, 119049 Moscow, Russia; 5 Department of Design and Manufacture of Radio Equipment, School of Electronic Engineering and Computer Science South Ural State University, 454080 Chelyabinsk, Russia; sv_truhanov@mail.ru (S.V.T.); max_v_333@mail.ru (M.G.V.); 86kds@mail.ru (D.S.K.); 6 Laboratory of Magnetic Films Physics, SSPA “Scientific and Practical Materials Research Centre of NAS of Belarus”, 220072 Minsk, Belarus; 7 Laser Research Group, Department of Physics & Center of Excellence in Nanotechnology, King Fahd University of Petroleum and Minerals (KFUPM), P.O. Box 5047, Dhahran 31261, Saudi Arabia; magondal@kfupm.edu.sa; 8 Center of Research Excellence in Nanotechnology (CENT), King Fahd University of Petroleum and Minerals (KFUPM), P.O. Box 5047, Dhahran 31261, Saudi Arabia; ashakeem@kfupm.edu.sa; 9 Laboratory “Electromagnetic Compatibility”, Centre of Collective Usage, Federal State Autonomous Educational Institution of Higher Education ‘Ural Federal University Named after the First President of Russia B.N. Yeltsin’, 620002 Ekaterinburg, Russia; 10 Department of Chemistry, Bharat Institute of Higher Education and Research (BIHER), 10 Bharat University, Chennai 600073, India; mkavath15@gmail.com; 11 Department of Nanomedicine Research, Institute for Research and Medical Consultations (IRMC), Imam Abdulrahman Bin Faisal University, P.O. Box 1982, Dammam 31441, Saudi Arabia; abaykal@iau.edu.sa

**Keywords:** hard/soft ferrite, nanocomposite, structural properties, magnetic properties, microwave absorption

## Abstract

This paper reports the correlation between the composition of the functional Sr_0.5_Ba_0.5_Sm_0.02_Fe_11.98_O_19_/*x*(Ni_0.8_Zn_0.2_Fe_2_O_4_) hard–soft nanocomposites (SrBaSmFe/*x*(NiZnFe) NCs), where 0.0 ≤ x ≤ 3.0, and their structural features, magnetic, and microwave properties. SrBaSmFe/*x*(NiZnFe) hard/soft ferrite NCs are produced using the *one-pot* citrate combustion method. According to the XRD analysis, all samples showed the co-existence of both SrBaSmFe and NiZnFe phases in different ratios. Magnetic properties are measured in a wide range of magnetic fields and temperatures (10 and 300 K) and correlated well with the composition of the investigated samples. The microwave properties (frequency dispersions of the magnetic permeability, and electrical permittivity) are discussed by using the co-axial method in the frequency range of 0.7–18 GHz. Non-linear dependences of the main microwave features were observed with varying of composition. The microwave behavior correlated well with the composite theory. These results could be used in practice for developing antenna materials.

## 1. Introduction

The development of new functional composites with controllable magnetic and microwave properties is one of the important tasks of modern applied physics. Nowadays, functional hard–soft magnetic composites are attracting the attention of scientists; spinel ferrites and composites based on them have had great contributions in many fields, such as materials for catalysis [1,2], targeted drug delivery [3], microwave, gas sensors [4], magneto-optical data storage [5], medicines, cancer treatment, telecommunications, photoactive materials, and photo-catalysts. Microwave absorption is one of the most promising directions of complex iron oxides [6,7] because its high saturation magnetization (M_s_), large coercivity (H_c_), and strong coupling between magnetic phases. This opens for new perspectives for the integration of the magnetic materials in electronics [8].

Kneller et al. [9] have proposed that permanent magnets could be produced based on composite materials comprising two appropriately dispersed hard and soft magnetic phases that are mutually exchange-coupled. The large coercivity (provided by hard magnetic phase) and the high saturation magnetization (provided by soft magnetic phase) of magnetic hard/soft ferrites composites can be achieved when the exchange coupling is performed [10,11,12,13]. Many researchers have reported the exchange coupling behavior for some multilayer and metal alloy systems like Nd–Fe–B [14], SmCo_x_–Co [15], Pr_2_Fe_12_B [16], and Sm–Co/Fe [17]. There are many kinds of hard/soft ferrite composites that have attracted the interest of researchers. For example, ferrite–polymer composites such as Ni_0.5_Zn_0.5_Fe_2_O_4_/BaFe_12_O_19_@polyaniline composites have been synthesized and investigated [6]. Additionally, the quite popular core-shell structures of NiFe_2_O_4_/SrCo_0.2_Fe_11.8_O_19_ [18] and Mn_0.6_Zn_0.4_Fe_2_O_4_@Sr_0.85_Ba_0.15_Fe_12_O_19_ [19] have been also explored. Ceramics-based composites such as SrTb_0.01_Tm_0.01_Fe_11.98_O_19_-AFe_2_O_4_ (A = Co, Ni, Zn, Cu and Mn) [20], BaFe_12_O_19_/CoFe_2_O_4_ [12], Sr_0.3_Ba_0.4_Pb_0.3_Fe_12_O_19_/(CuFe_2_O_4_)_x_ [7] (Ba_0.5_Sr_0.5_Fe_12_O_19_)_1−x_(CoFe_2_O_4_)_x_ [21], SrFe_12_O_19_/Ni_0.7_Zn_0.3_Fe_2_O_4_ [22], Li_0.3_Co_0.5_Zn_0.2_Fe_2_O_4_/SrFe_12_O_19_ [23], and fiber-based composites such as SrFe_12_O_19_/Ni_0.5_Zn_0.5_Fe_2_O_4_ nanofibers [11] have been intensively investigated. Great attention has been paid to hard/soft magnetic nanocomposites (NCs) due to the significant improvement of their overall magnetic properties. It has been verified that the composition, microstructure, grain size, and strength of magnetic interaction greatly affect the exchange coupling between hard and soft ferrite phases [7,12]. The structural and magnetic properties of these NCs could be improved by optimal calcination conditions, appropriate hard–soft ratios, and well-exchange coupling between hard and soft ferrite phases [23,24,25,26]. The achievement of well-exchange coupling in hard/soft ferrite nanocomposites is still a challenging task to be accomplished [27]. Accordingly, it is very important to synthesize composite materials that display well-exchange coupling behavior [28,29,30].

Separate hard and soft ferrites are favorable for outstanding applications [31]. Soft spinel ferrites with a low anisotropic value are suitable for, e.g., microwave applications [32]. On the other hand, hard ferrites such as BaFe_12_O_19_ have a high ferromagnetic resonance frequency (~35 GHz) and a high magnetocrystalline anisotropy, making them suitable for W-band applications [33]. Consequently, combinations of low anisotropic soft ferrite and high anisotropic hard ferrite are largely used as permanent magnets, as well as for different microwave device applications such as functional devices (circulators and inventors) and antennae [34,35]. Radar absorbing materials (RAM) have excellent microwave absorbing properties due to their high range of 8.2–12.4 GHz [36]. However, a large absorption peak, thin absorption layer, and wide working frequency range cannot be achieved by using a single material of an ideal radar absorber [8,37]. Nanocomposites could help to construct unified systems comprising hard and soft ferrite phases, where the properties would be exclusive and complimentary [21]. Magneto-dielectric composites have a high permeability and a highly permittie nature due to the exchange coupling effect that could substitute dielectric substrates for antenna miniaturization. However, some composites are scarcely used because of their high frequency range between 12 and 18 GHz [38].

There are two ways to develop hard–soft ferrite nanocomposites. One of them is concerned with varying the chemical composition of the initial components. The second one is concerned with varying the mass ratio of soft and hard phases. In this study, SrBaSmFe/*x*(NiZnFe) hard/soft ferrite NCs with varying hard/soft phases ratios of 1:x (where 0.0 ≤ x ≤ 3.0) were synthesized. The structural, magnetic, and microwave properties were deeply investigated. The correlation between composition, structural features, microwave properties, and magnetic properties of the composites is discussed in terms of coupling strength between different constituting phases.

## 2. Materials and Methods 

**Figure 1:**
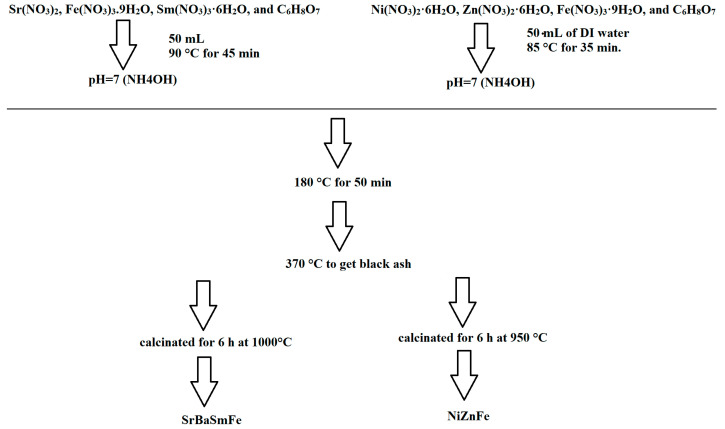
Synthesis stages of SrBaSmFe/*x*(NiZnFe) (0.0 ≤ x ≤ 3.0) nanocomposites (NCs).

Hard/soft ferrite SrBaSmFe/*x*(NiZnFe) (0.0 ≤ x ≤ 3.0) NCs were prepared via the *one-pot* citrate combustion methodology, as shown in Figure 1 [39]. Firstly, SrBaSmFe and NiZnFe were prepared individually through the sol–gel auto-combustion approach. In order to prepare SrBaSmFe, a specific amount of Sr(NO_3_)_2_, Fe(NO_3_)_3_.9H_2_O, Sm(NO_3_)_3_·6H_2_O, and C_6_H_8_O_7_ were dissolved in 50 mL of deionized (DI) water under continuous stirring at 90 °C for 45 min to get the SrBaSmFe solution. For the particular synthesis of the NiZnFe solution, Ni(NO_3_)_2_·6H_2_O, Zn(NO_3_)_2_·6H_2_O, Fe(NO_3_)_3_·9H_2_O, and C_6_H_8_O_7_ were dissolved together in 50 mL of DI water under stirring at 85 °C for 45 min. The pH of both solutions was regulated at 7 with an ammonium hydroxide solution (NH_4_OH) while tuning the temperature to 180 °C for 50 min and then raising it to 370 °C to get black ash. The final products of SrBaSmFe and NiZnFe were calcinated for 6 h at 1000 and 950 °C, respectively.

The SrBaSmFe/NiZnFe hard/soft ferrite NCs with different fractions were produced by mixing the initial solutions of SrBaSmFe and NiZnFe under continuous stirring at 85 °C for 35 min. The pH of the hard/soft solution was modified with an ammonium solution to reach 7. At that time, the temperature was increased to 180 °C for 50 min and then raised again to 350 °C until a black powder was obtained. The resulting powders were calcinated at 1000 °C for 6 h to get the final hard/soft nanocomposites.

The hard/soft ferrite NC structure was examined by employing XRD (X-ray powder diffraction) Rigaku D/MAX-2400 (Cu K*α*). The morphology was observed via FE-SEM (Lyra3, Tescan, Brno, Czech Republic) coupled with an energy-dispersive X-ray (EDX) system. Transmission electron microscope (TEM) and high-resolution transmission electron microscope (HR-TEM) (FEI Titan ST Microscopes) were employed to approve the morphology and structure. A vibrating sample magnetometer (VSM) was used to get the magnetic measurements of the products. Microwave parameters (permittivity and permeability) were estimated as frequency dispersions of the real and imaginary parts from direct S-parameters measurements. The S-parameters of the transmission line were analyzed by means of a vector network analyzer (R&S model ZVA24) in the 8–12 GHz range. S11 parameters were measured in two regimes: Firstly, in a short circuit regime and secondly, in a matched-load regime. The measured values were used to calculate the frequency dispersions of the magnetic permeability and electrical permittivity (real and imaginary parts). The measurement procedure was reported in [40]. Full two-port calibration was initially performed on the test setup to remove errors due to the directivity, source match, load match, isolation, and frequency response in both the forward and reverse measurements. The measured reflection coefficient (S11) and transmission coefficient (S21) of the samples were converted into real and imaginary parts of permeability; the permittivity (${\dot{\mu }}^{\prime }$ and ${\dot{\mu }}^{\prime \prime }$) and permeability (${\dot{\varepsilon }}^{\prime }$ and ${\dot{\varepsilon }}^{\prime \prime }$) of the material were calculated by the Nicholson–Ross–Weir algorithm from the S-parameters recorded as a function of frequency.

## 3. Results and Discussion

The main idea of the conducted research was the observation and explanation of the correlation between composition (ratio between soft and hard phases), crystal structure, microstructural features, magnetic properties, and microwave properties in the composites. The correlation between structural parameters and magnetic properties for both magnetic phases and the existence of outstanding magnetic exchange-coupling between the phases could open perspectives for the observation of any phenomena in magnetic and microwave properties.

### 3.1. Microstructure

**Figure 2:**
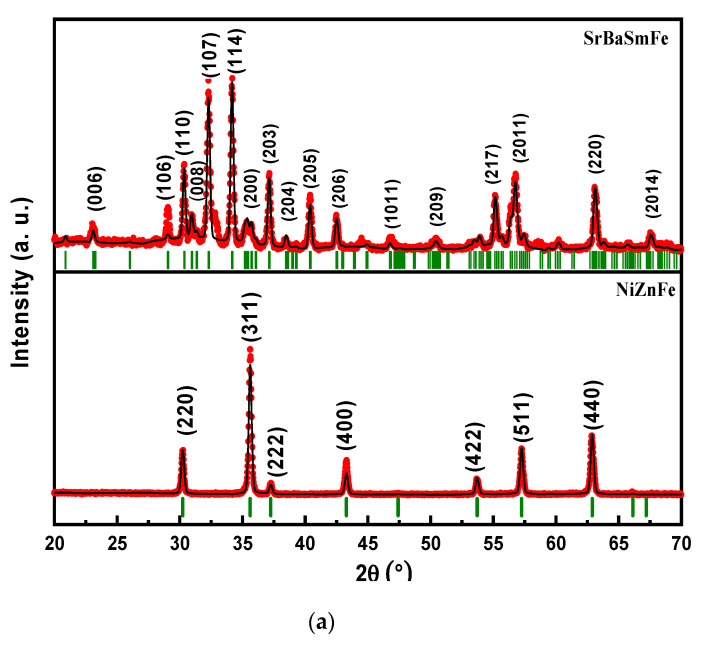

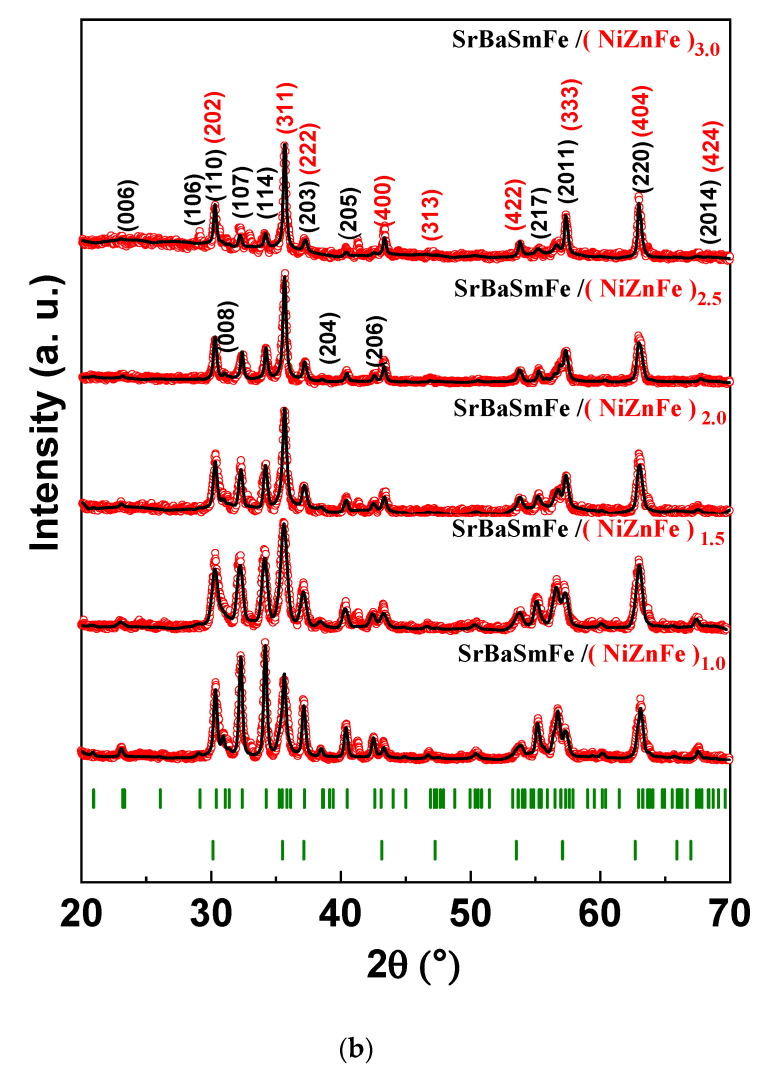
XRD powder patterns of (**a**) pure SrBaSmFe, NiZnFe, and (**b**) SrBaSmFe/(NiZnFe)_x_ hard/soft ferrite nanocomposites (1 ≤ x ≤ 3).

The structural analyses of SrBaSmFe/*x*(NiZnFe) (0.0 ≤ x ≤ 3.0) hard/soft ferrite NCs were implemented through the investigation of XRD powder patterns. Figure 2a presents the XRD patterns of pure SrBaSmFe and NiZnFe separately. These patterns show the characteristics peaks of M-type hexaferrite for the SrBaSmFe sample and a cubic spinel structure for the NiZnFe product. No undesired impurity was observed in either sample. On the other hand, Figure 2b presents the XRD patterns of various hard/soft SrBaSmFe/x(NiZnFe) NCs where *x* = 1.0–3.0. XRD patterns for all compositions showed the successful co-formation of M-type hexaferrite and cubic spinel phases within the nanocomposite, and no impurity was discerned. It is obvious that there is a disparity in the intensity of indexed peaks of hard/soft ferrite NCs because of the gradual increase in the spinel content. This can be attributed to the effectiveness of the synthesis method, which allowed for homogenization between the hexagonal and spinel phases. The structural parameters and the fraction percentage of various hard/soft ferrite SrBaSmFe/*x*(NiZnFe) (0.0 ≤ x ≤ 3.0) NCs were determined via Match 3! and Full Proof software (Table 1). It was noticed that the lattice constant ‘*a’* of the hard ferrites increased with the addition of soft ferrite, while the lattice parameter ‘*c’* of hard ferrite and ‘*a’* of soft ferrite fluctuated, maybe due to the alteration in the solubility between the soft and hard ferrite [41]. The average crystallite sizes (D) of SrBaSmFe and NiZnFe were calculated by applying the Scherrer equation (D_XRD_ = Kλ/βcosθ, where K is a shape constant, λ is the wavelength of CuKα radiation (1.5406 Å), and β is the peak width at half maximum intensity) on the intense peaks of hard and soft ferrites. It was noticed that the crystallite size of the hard phase was around 45% larger than that of the soft phase. The crystallites size varied from 50 to 83 nm and from 21 to 50 nm for the hard and soft products, respectively.

### 3.2. FESEM and TEM Analysis

**Figure 3:**
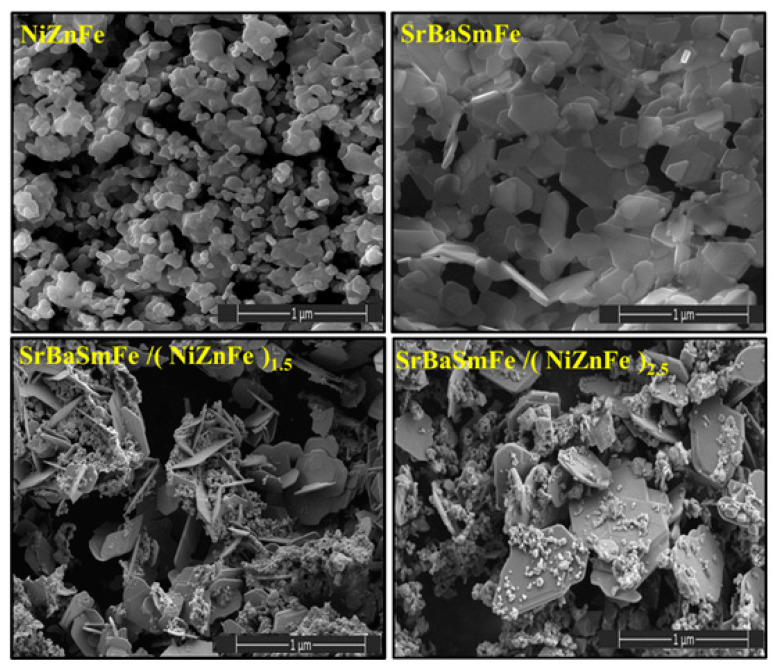
FE-SEM images of pure SrBaSmFe, pure NiZnFe, SrBaSmFe/(NiZnFe)_1.5_ NC, and SrBaSmFe/(NiZnFe)_2.5_ NC.

**Figure 4:**
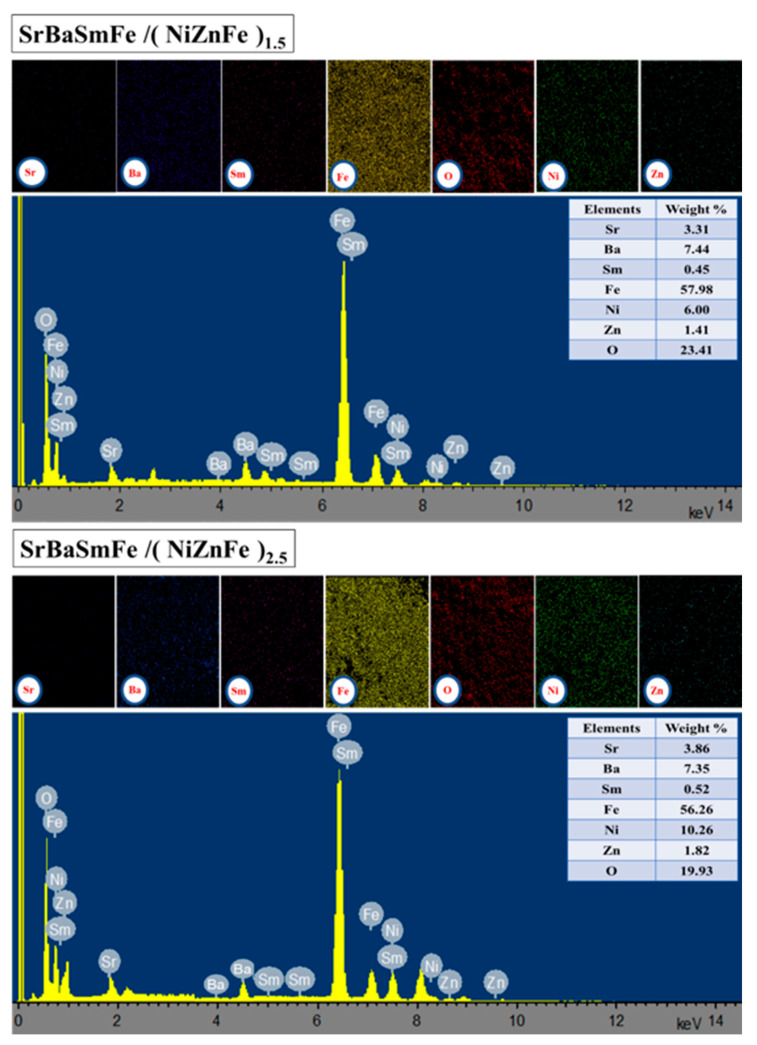
Energy-dispersive X-ray (EDX) and elemental mapping of hard/soft ferrite SrBaSmFe/(NiZnFe)_x_ NCs with *x* = 1.5 and 2.5.

**Figure 5:**
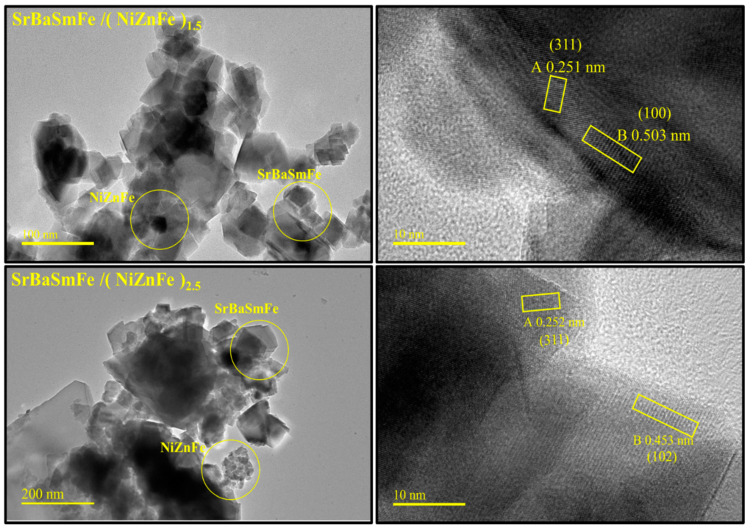
TEM (left) and HR-TEM (right) images of hard/soft ferrite SrBaSmFe/(NiZnFe)_x_ NCs with *x* = 1.5 and 2.5.

The analyses of the microstructure of pure SrBaSmFe, pure NiZnFe, and various hard/soft ferrite NCs of SrBaSmFe/x(NiZnFe), where *x* = 1.5 and 2.5, were performed with the FE-SEM technique (Figure 3). The images revealed that the morphology of NiZnFe consisted of aggregated grains with spherical shapes. Meanwhile, the SrBaSmFe NCs exhibited randomly oriented hexagonal platelets grains. The FE-SEM images of hard/soft ferrite SrBaSmFe/*x*(NiZnFe) NCs with *x* = 1.5 and 2.5 disclosed hexagonal plates decorated by clusters of spherical grains. It was obvious that the morphology of hard/soft ferrite NCs was changed upon the increase of the content of spinel ferrite to become most hexagonal platelets covered by assembled spherical grains. The EDX and elemental mapping of hard/soft ferrite SrBaSmFe/*x*(NiZnFe) NCs with *x* = 1.5 and 2.5 were carried out and are presented in Figure 4. Theses analyses showed the existence of various required elements and verified the successful formation of the desired compositions. This proved the efficiency of the used preparation method. To further confirm the formation and the morphology of hard/soft ferrite SrBaSmFe/*x*(NiZnFe) NCs, TEM, and HR-TEM investigations for *x* = 1.5 and 2.5 NCs were performed (Figure 5). The TEM images demonstrated the combination of hard and soft phases. The interplanar fringes provided by HR-TEM images were consistent with the hard and soft phases, which confirmed the coexistence of both hard and soft phases.

### 3.3. VSM Investigation

**Figure 6:**
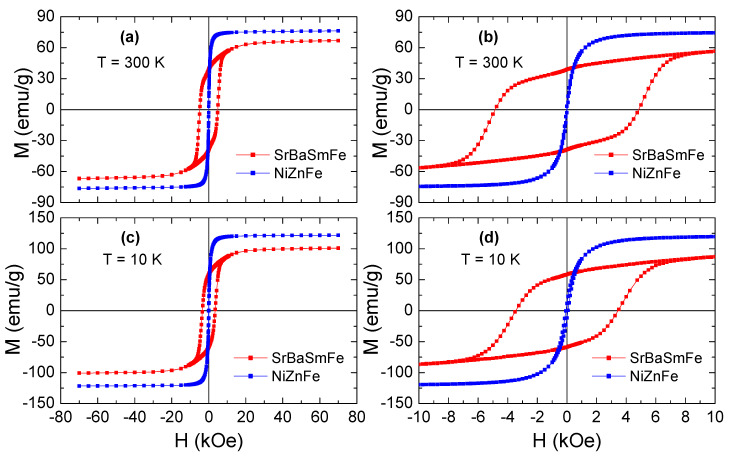
M(H) hysteresis curves recorded at (**a**,**b**) T = 300 K and (**c**,**d**) T = 10 K for Ni_0.8_Zn_0.2_Fe_2_O_4_ (NiZnFe) and Sr_0.5_Ba_0.5_Sm_0.04_Fe_11.96_O_19_ (SrBaSmFe) samples. (**b**) and (**d**) are enlarged views of magnetic hysteresis loops.

Figure 6 provides the variations of magnetization (M) as a function of an applied magnetic field (H), as found by exposing samples of SrBaSmFe and NiZnFe to a magnetic field H = ±70 kOe. The magnetic measurements were carried out at two measured temperatures of T = 300 and 10 K. The M(H) plots of pure NiZnFe nanoparticles (NPs) showed roughly S-shaped behavior without coercivity (H_c_) and remnant magnetization (M_r_), thus revealing that these NPs were superparamagnetic (SPM) at both temperatures. The M_s_ values for NiZnFe NPs were about 76.6 and 121.9 K at 300 and 10 K, respectively. However, pure SrBaSmFe hexaferrite (HF) displayed ferrimagnetic (FM) behavior at both 300 and 10 K. Indeed, SrBaSmFe HFs disclosed *H_c_* values of 4852 and 3486 Oe at 300 and 10 K, respectively, and its M_s_ and M_r_ values were about 67.0 and 39.6 emu/g at 300 K and around 101.6 and 59.4 emu/g at 10 K. The very high M_s_ values of soft magnetic NiZnFe nano-spinel ferrites and the large coercivity of hard magnetic SrBaSmFe HFs could suggest that combining these two phases would be appropriate for developing well exchange-coupled nanocomposites.

**Figure 7:**
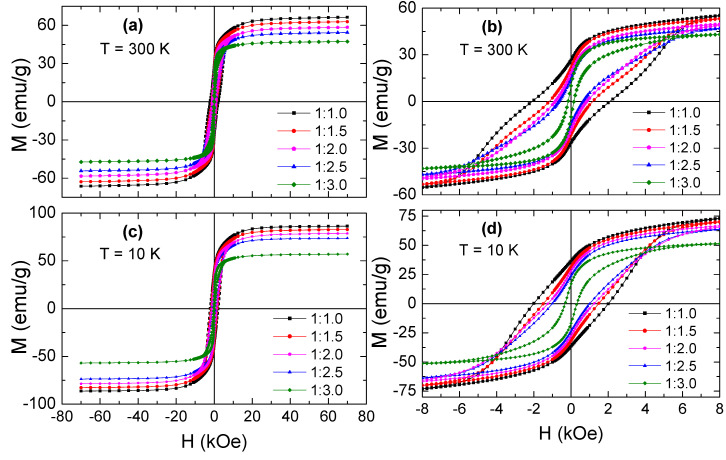
M(H) hysteresis curves performed at (**a**,**b**) T = 300 K and (**c**,**d**) T = 10 K for hard/soft ferrite SrBaSmFe/(NiZnFe)_x_ NCs (1.0 ≤ x ≤ 3.0) products.

In this work, the exchange-coupling behavior in the produced products of Sr_0.5_Ba_0.5_Sm_0.04_Fe_11.96_O_19_/*x* Ni_0.8_Zn_0.2_Fe_2_O_4_ (or SrBaSmFe/*x*NiZnFe for brevity), where *x* content is equal to 0.0–3.0, were carefully investigated through VSM measurements. Figure 7 presents the M(H) curves of various produced products by exposing them to H = ±70 kOe at 300 K and 10 K. All produced products exhibited FM characteristics at both 300 and 10 K. M_s_ magnitudes were in the interval of 47.3–66.3 emu/g at 300 K and of 57.1–86.4 emu/g at 10 K. M_r_ values were in the interval of 10.1–26.7 emu/g at 300 K and of 17.0–36.6 emu/g at 10 K. H_c_ magnitudes ranged between 155 and 2103 Oe at 300 K and between 319 and 2158 Oe at 10 K. The magnetizations were increased for various prepared products at 10 K in comparison to those at 300 K. In fact, once a negative impact of thermal energy on the quantitative organization of magnetic moments considerably reduces, the recorded magnetization certainly increases [42,43]. This largely eminent aspect that is evidenced from the various M(H) hysteresis loops of produced products is their shapes. Indeed, the different produced products illustrated the presence of a “kink” in the variation of M(H) curves, as displayed in Figure 7b,d. Such an observed kink reflects uncompleted exchange coupling among the two phases; hence, the hard and soft phases are separately switching [44]. Consequently, an overlapping of two loops resulting from separated soft and hard phases will be perceived [45].

**Figure 8:**
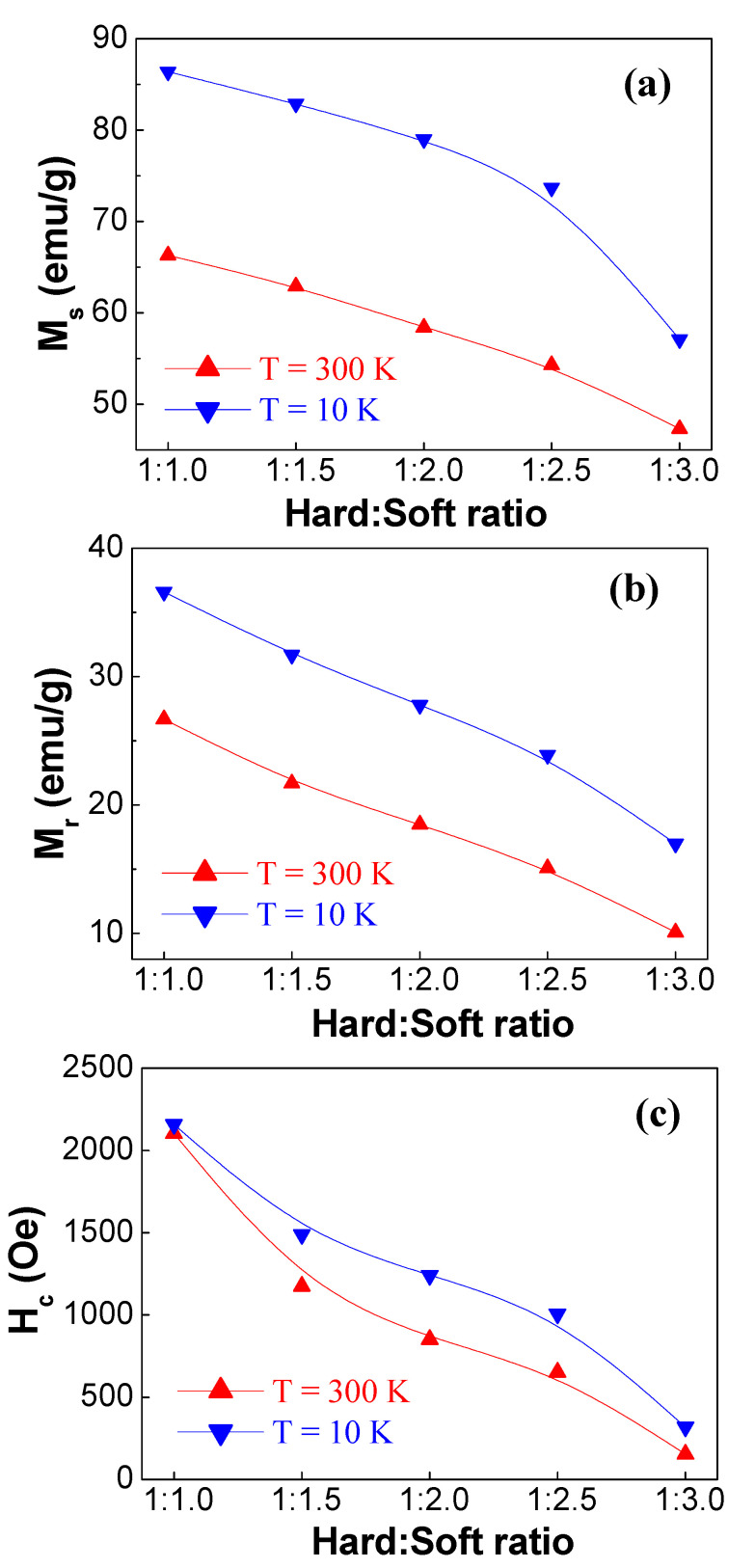
Variations in (**a**) saturation magnetization (M_s_), (**b**) remnant magnetization (M_r_), and (**c**) coercivity (H_c_) at both 300 and 10 K for various produced hard/soft ferrite SrBaSmFe/(NiZnFe)_x_ NCs (1.0 ≤ x ≤ 3.0) products.

The different magnetic parameters were deduced for various products. For better presentation, the progressions in M_r_, M_s_, and H_c_ values are presented against the various amounts of the NiZnFe soft phase in Figure 8. The M_s_ values were estimated from the extrapolation of M against 1/H^2^ plots, as reported in the following references [46,47,48]. At both 300 and 10 K, the highest M_s_, M_r_, and H_c_ magnitudes belonged to SrBaSmFe/1.0(NiZnFe) product (i.e., *x* = 1.0) and continuously reduced with rising the weight fraction of the NiZnFe soft phase. For this product, M_s,max_ and M_r,max_ were equal to 66.3 and 26.7 emu/g at 300 K and 86.4 and 36.6 emu/g at 10 K, respectively. The larger H_c_ magnitudes were about 2103 and 2158 Oe at 300 and 10 K, respectively. The smallest M_s_, M_r_, and H_c_ parameters were registered for SrBaSmFe/3.0(NiZnFe) product (i.e., *x* = 3.0). This nanocomposite sample had M_s_ = 47.3 and 57.1 emu/g at room temperature (RT) and 10 K, respectively. M_r,min_ were nearly 10.1 and 17.0 emu/g at RT and 10 K, respectively. H_c_ reduced sharply to values of 155 and 319 Oe for the *x* = 3.0 product. Generally, two chief interactions prevail in hard/soft products; those among hard/hard grains and soft/soft grains that are referred as dipolar interactions and those between hard/soft grains that are denoted as exchange-coupling interactions [49,50]. When the dipolar interactions are irrelevant, the magnetization is chiefly governed by exchange interactions and magneto-crystalline anisotropy. Because of rising the soft composition within the products, the dipolar interactions come to be more important. As consequence, the M_s_, M_r_, and H_c_ values of products will diminish.

The magneton numbers ($n_{B}$) were determined for hard SrBaSmFe and soft NiZnFe phases from the common formula $n_{B}=molecular\;weight\times M_{s}/5585$ [51]. Magneton numbers were calculated as 3.22 μ_B_ (at 300 K) and 5.12 μ_B_ (at 10 K) for the NiZnFe soft spinel ferrite. On the other hand, $n_{B}$ values were found to be around 13.06 and 19.80 μ_B_ for SrBaSmFe HFs at 300 and 10 K, respectively. Nevertheless, this simple formula could not be used for the produced products since they comprised the contributions of resultant magnetic moments coupled with the fractions (f_MW_) of NiZnFe and SrBaSmFe phases in the products. Accordingly, to determine accurately the total magneton numbers for the produced products, the following relation was employed:

(1) $$n_{B}=f_{MW,NiZnFe}*n_{B,NiZnFe}+\left(1-f_{MW,NiZnFe}\right)*n_{B,SrBaSmFe}$$

At RT, the determined magneton number decreased linearly from a maximum value $n_{B}=8.14\;\mu_{B}$ that belonged to the SrBaSmFe/(NiZnFe)_1.0_ product to a minimum value $n_{B}=5.68\;\mu_{B}$ for the SrBaSmFe/(NiZnFe)_3.0_ product. At T = 10 K, $n_{B}$ magnitude decreased with the increasing NiZnFe fractions; it decreased from 12.46 in the *x* = 1.0 product to 8.79 μ_B_ in the *x* = 3.0 NC product. The observed tendency in the variations of magneton numbers was in line with the observed tendency of M_s_ and M_r_ values with respect to the NiZnFe content.

The squareness ratios (SQR) = *M*_r_/*M*_s_ were calculated (Table 2). An SQR can give information about the magnetic domains of a nanoparticle system. A theoretically predicted value above 0.50 is accredited to a single-domain structure [46]. However, an SQR < 0.5 is assigned to a multi-domain nature. In the present study, the SQR decreased from 0.403 to 0.214 at 300 K and from 0.424 to 0.298 at 10 K with the increase of the NiZnFe fraction in the produced products. All these values were below 0.5, which suggests a multi-magnetic domain nature for all products at both 300 and 10 K.

**Figure 9:**
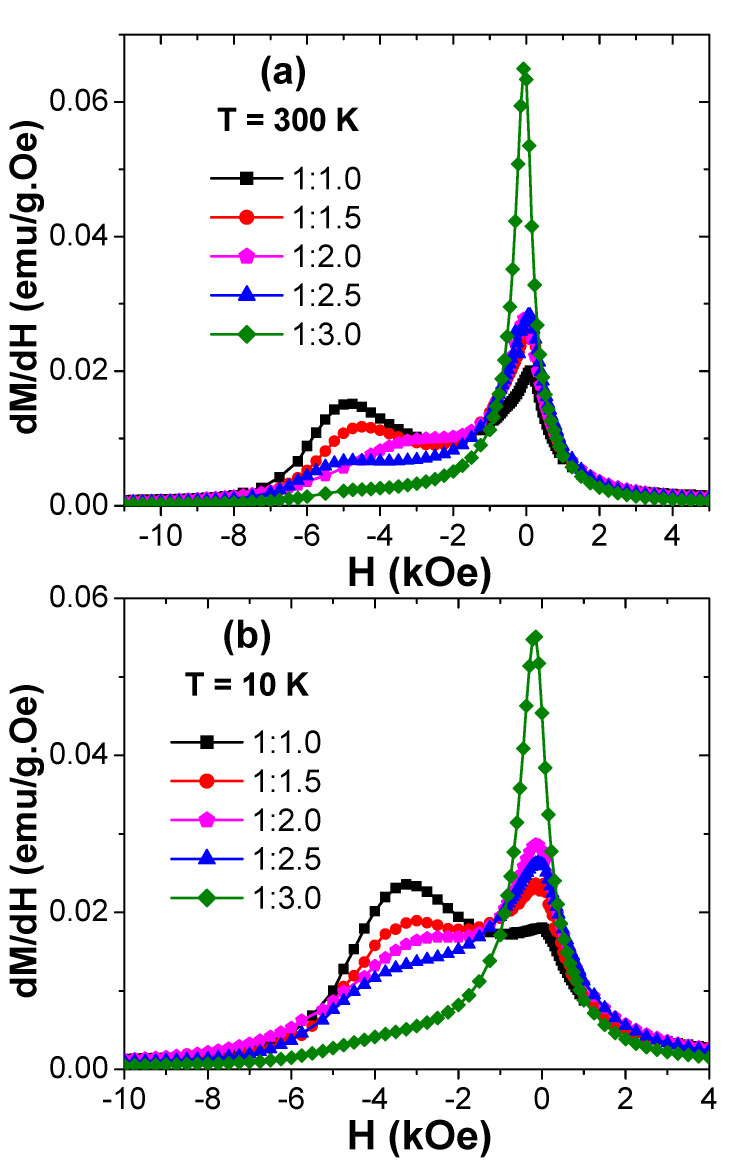
*dM/dH* vs. *H* plots performed at (**a**) T = 300 K and (**b**) T = 10 K for various prepared hard/soft ferrite SrBaSmFe/(NiZnFe)_x_ NCs (0 ≤ x ≤ 3) products.

To further evaluate the effect of exchange-coupling within the produced products, the curves of the derivative of magnetization per applied magnetic field (d*M*/d*H*) against field were investigated (Figure 9) [52,53]. Typically, there will be a singular peak once the exchange coupling between the two phases is achieved. Nevertheless, binary separate maxima occur if the exchange coupling is still not completed [54]. In our case, two dissimilar peaks were observed in diverse products, illuminating that the reversal magnetization was unachievable by one-stage. These two distinctive peaks in d*M*/d*H* curves suggested that the magnetic spins of the soft and hard phases were switching individually.

### 3.4. Microwave Properties

**Figure 10:**
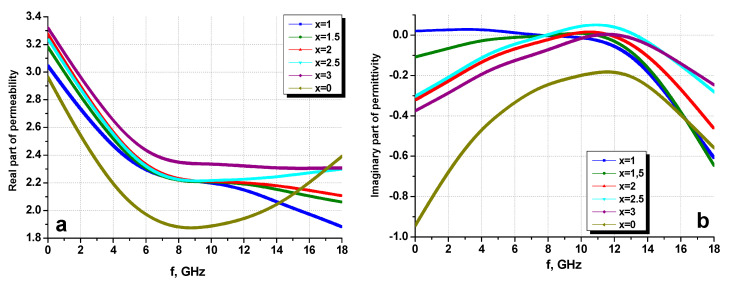
Frequency dispersions of the real part (**a**) and imaginary part (**b**) of permittivity for the SrBaSmFe/(NiZnFe)_x_ NCs.

Using the co-axial method (transmission line), measurements of the magnetic permeability and electrical permittivity (frequency dispersions of their real and imaginary parts) of the SrBaSmFe/*x*(NiZnFe) hard/soft ferrite NCs with varying hard/soft phases ratio 1:x (0.0 ≤ x ≤ 3.0) were performed. Figure 10 shows the dependences of the real (Figure 10a) and imaginary (Figure 10b) parts of the permittivity as a function of the frequency of the all composites ratio. It is clear that the chemical content (concentration of the soft phase-x) significantly affected the value of permittivity.

The obtained dispersions for the SrBaSmFe/*x*(NiZnFe) hard/soft ferrite NCs were in good agreement with the frequency dependences obtained from the standard theory for microwave properties in composites [40]. From the obtained results, it was clear that sample No. 6 did not have standart behavior. Since the various prepared hard/soft ferrite NCs were composed of two materials (Sr_0.5_Ba_0.5_Sm_0.02_Fe_11.98_O_4_ hard and Ni_0.8_Zn_0.2_Fe_2_O_4_ soft phases), the dielectric constant of these materials at frequencies from 1 to 14 GHz was from 4 to 5, and the value of the real part of the electrical permittivity of the composites practically did not vary with increasing frequency.

Only at frequencies above 14 GHz did the dependencies take on different values in frequency. For the imaginary part of the permittivity, the dependences of the materials were different: the losses in Sr_0.5_Ba_0.5_Sm_0.02_Fe_11.98_O_4_ were higher, and, therefore, the nature of the change in values was such that with an increase in the number of spinel, the graph of the frequency dependence mixed down along the *y*-axis.

With dipole polarization, the losses in the dielectric were minimal; therefore, the imaginary part of the permittivity did not vary with increasing frequency; see Figure 10. A significant decrease in the imaginary permittivity and an increase of losses were because, at these frequencies, a transition began from dipole to electronic polarization. This was due to the orientation of electrons in the electric field.

**Figure 11:**
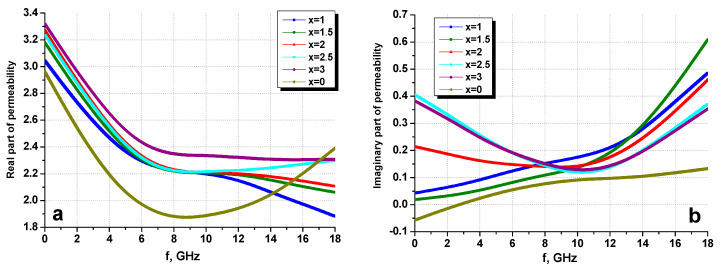
Frequency dispersions of the real part (**a**) and imaginary part (**b**) of permeability for the SrBaSmFe/(NiZnFe)_x_ NCs.

Figure 11 shows the dependences of the real (Figure 11a) and imaginary (Figure 11b) parts of the permeability as a function of the frequency of the all composites ratio.

For the real part of magnetic permeability, the values for hexaferrite were smaller (dependence of 6 at the very bottom) than those of the spinel; therefore, with an increase for spinel, the dependence graph mixed up along the *y*-axis. Theoretically, if pure spinel was measured, the dependences for composites can be predicted.

## 4. Conclusions

Functional hard/soft ferrite NCs (with the chemical formula of Sr_0.5_Ba_0.5_Sm_0.02_Fe_11.98_O_4_/x(Ni_0.8_Zn_0.2_Fe_2_O_4_)) (0.0 ≤ x ≤ 3.0) were fabricated by the *one-pot* citrate combustion method. According to the XRD results, all samples only contained initial SrBaSmFe and NiZnFe components in different ratios. The microstructure of the composite samples correlated with the shape and size of the initial components and their ratios. The morphology of hard, soft, and hard/soft NCs revealed three categories of particle shapes such as hexagonal plate, spherical, and an assembly of both hexagonal and spherical. M(H) hysteresis loops showed the hard ferrimagnetic nature of various produced products at both 300 and 10 K. The hard FM behavior of SrBaSmFe was considerably altered by raising the fraction of soft NiZnFe in the mixtures. The magnetization values (M_s_, M_r_, and H_c_) were the highest for the SrBaSmFe/1.0(NiZnFe) hard/soft ferrite product (i.e., *x* = 1.0) and continuously reduced when raising the weight fraction of the NiZnFe soft phase. Microwave properties such as the frequency dispersions of the magnetic permeability and electrical permittivity (their real and imaginary parts) were measured with the co-axial method in the 1–18 GHz range. At these frequencies, the main mechanism contributing to the dielectric constant was dipole polarization. In the dipole polarization model, the process of orientation of the dipoles may not have time to induce changes in the external field; due to this, with increasing frequency, the real electrical permittivity decreased with increasing frequency. The real and imaginary magnetic permeabilities let us conclude that the behavior is typical for ferrimagnetics This means that the main losses in this type of composites can be attributed to electrical losses (dipole polarization).

## Figures and Tables

**Table 1:** Crystallites size (D_XRD_), lattice parameters, and phase fractions calculated for both hard (107 plane) and soft (311 plane) phases in hard/soft ferrite nanocomposites of SrBaSmFe/(NiZnFe)_x_ (0 ≤ x ≤ 3).

Nanocomposite	D_XRD_ (nm)		Hard Phase			Soft Phase	
	Soft	Hard	a = b (Å)	c (Å)	Fraction (%)	a = b = c (Å)	Fraction (%)
SrBaSmFe	-	50.9	5.8845	23.1037	100	-	-
SrBaSmFe/(NiZnFe)_1.0_	21.5	48.3	5.8868	23.0963	81.1	8.3591	18.9
SrBaSmFe/(NiZnFe)_1.5_	26.1	37.8	5.8878	23.1177	64.2	8.3581	35.8
SrBaSmFe/(NiZnFe)_2.0_	27.7	55.7	5.8948	23.1152	53.3	8.3536	46.7
SrBaSmFe/(NiZnFe)_2.5_	30.4	49.9	5.8950	23.1342	46.2	8.3521	53.8
SrBaSmFe/(NiZnFe)_3.0_	50.3	82.9	5.9002	23.0512	29.0	8.3439	71.0
NiZnFe	44.1	-	-	-	-	8.3553	100

**Table 2:** Squareness ratio (SQR) and magneton numbers (*n_B_*) of the hard/soft ferrite products of SrBaSmFe/(NiZnFe)_x_ NCs where *x* = 1.0 up to 3.0.

H:S Ratio	SQR		*n_B_* (*μB*)	
	300 K	10 K	300 K	10 K
1:1.0	0.403	0.424	8.14	12.46
1:1.5	0.345	0.382	7.15	10.99
1:2.0	0.317	0.352	6.50	10.01
1:2.5	0.278	0.324	6.03	9.31
1:3.0	0.214	0.298	5.68	8.79
